# An Optimized Approach for Neuron‐Like Differentiation of SH‐SY5Y Neuroblastoma Cells Using Retinoic Acid and a Laminin‐Rich Extracellular Matrix

**DOI:** 10.1002/alz70856_105542

**Published:** 2026-01-07

**Authors:** Marina Mantellatto Grigoli, Bianca Cruz Pachane, Angelina Maria Fuzer, Sabrina Dorta de Oliveira, Ana Beatriz Aparecida Targas, Vanessa Alexandre‐Silva, Heloisa Sobreiro Selistre‐de‐Araujo, Patricia Regina Manzine, Marcia Regina Cominetti

**Affiliations:** ^1^ Federal University of São Carlos, São Carlos, São Paulo, Brazil; ^2^ Federal University of São Carlos, São Carlos, SP, Brazil; ^3^ Global Brain Health Institute, Dublin, Dublin, Ireland; ^4^ The Global Brain Health Institute, Trinity College Dublin, Dublin, Dublin, Ireland

## Abstract

**Background:**

The SH‐SY5Y neuroblastoma cell line is a valuable in vitro model for studying neuronal differentiation and neurodegenerative diseases like Alzheimer's disease (AD). Traditional differentiation protocols mainly use retinoic acid (RA); however, they lack extracellular matrix (ECM) components that are critical for mechanotransduction and cellular adhesion, which limits their physiological relevance. Laminins, a key ECM glycoprotein, play an essential role in neurite outgrowth and synaptic formation, indicating their potential to enhance neuronal differentiation.

**Method:**

SH‐SY5Y cells were cultured in DMEM/F12 supplemented with fetal bovine serum (FBS) and essential additives. Differentiation was induced using RA (10 µM and 25 µM) and a laminin‐rich ECM (LrECM). Plates were pre‐coated with Matrigel® (a laminin‐rich ECM) before seeding the cells. Differentiation efficiency was monitored over 10 days through light microscopy, immunofluorescence for neuronal markers (NeuN and β3‐tubulin), and acetylcholinesterase (AChE) activity assays. Western blotting assessed β3‐tubulin expression, and neurite lengths were quantified using FIJI software.

**Result:**

The combined RA and LrECM treatment significantly enhanced SH‐SY5Y differentiation when compared to RA alone. Neuronal morphology, marked by extensive neurite outgrowth, became evident as early as day 4 and was sustained for up to 10 days. Immunofluorescence confirmed increased NeuN expression, showing a shift from cytoplasmic to perinuclear localization over time. β3‐tubulin levels remained consistently high in LrECM‐treated cells, unlike those treated with RA alone, which demonstrated a decline after day 7. Enhanced cholinergic differentiation was indicated by elevated AChE activity, particularly at 25 µM RA, although higher RA concentrations were unable to sustain neuronal characteristics and raised concerns about cytotoxicity.

**Conclusion:**

The incorporation of LrECM into SH‐SY5Y differentiation protocols significantly enhances neuronal differentiation and maintains neuron‐like characteristics, providing a more physiologically relevant in vitro model for studying AD and other neurodegenerative diseases. This approach enables cost‐effective, rapid differentiation and more accurately mimics the brain microenvironment, establishing a strong platform for neurobiological research and therapeutic screening.

